# Research on a Lightweight Recognition Model for Daily Cattle Behavior Toward Real-Time Monitoring

**DOI:** 10.3390/vetsci12121166

**Published:** 2025-12-08

**Authors:** Jianping Yao, Yong’an Zhang, Mei’an Li, Jia Li, Yanqiu Liu, Feilong Kang, Fan Liu

**Affiliations:** 1 College of Computer and Information Engineering, Inner Mongolia Agricultural University, Hohhot 010018, China; yaojianping@emails.imau.edu.cn (J.Y.); 20071036@imau.edu.cn (M.L.); lijia@imau.edu.cn (J.L.); liuyq@imau.edu.cn (Y.L.); lfmw@emails.imau.edu.cn (F.L.); 2 College of Mechanical and Electrical Engineering, Inner Mongolia Agricultural University, Hohhot 010018, China

**Keywords:** computer vision, cattle behavior, lightweight model, edge computing, animal welfare

## Abstract

Accurate monitoring of cattle behavioral time budgets is crucial for early disease detection and welfare assessment. Changes in durations of standing, lying, and eating are known to be early indicators of health issues such as lameness and metabolic disorders. To enable low-cost, non-invasive, and real-time monitoring, this study proposes a lightweight cattle behavior recognition method based on an improved YOLO11n architecture. The model enhances multi-scale feature integration through a generalized efficient layer aggregation network (GELAN), improves feature extraction via a multidimensional collaborative attention (MCA) mechanism, and achieves efficient cross-scale fusion using a bidirectional feature pyramid network (BiFPN). Depthwise separable convolution (DWConv) is incorporated to reduce computational load. Experimental results demonstrate high recognition accuracy, with mAP@0.5 values of 91.2%, 91.0%, and 93.9% for standing, lying, and eating, respectively. The model was subsequently compressed using a Layer-adaptive Magnitude-based Pruning (LAMP) algorithm, resulting in a final model of only 1.06 × 10^6^ parameters, a computational cost of 6.3 GFLOPS, and a weight size of 2.4 MB, while retaining 90.7% mAP@0.5. This highly efficient system is suitable for deployment on resource-constrained edge devices, providing a practical tool for continuous cattle monitoring. It offers a viable pathway for farmers to adopt precision livestock farming practices, facilitating early health intervention and promoting animal welfare.

## Simple Summary

Effectively monitoring the daily behaviors of cattle, such as standing, lying down, and feeding, is crucial for farmers to ensure animal health and welfare. However, manual observation is time-consuming and impractical for continuous monitoring. While some automated methods use wearable sensors, these can be expensive, stressful for the animals, and difficult to maintain. This study aimed to provide a low-cost, efficient solution using common farm cameras. We developed a smart computer vision model that can automatically identify these key cattle behaviors from video footage. The model was specially designed to be compact and fast, allowing it to run on simple, low-power computing devices often used on farms. After testing, our model achieved high accuracy in recognizing all three behaviors. In addition, its small size and low processing demands mean it can provide real-time monitoring without expensive equipment. This offers a practical and affordable tool for small and medium-sized farms to adopt intelligent management practices, helping farmers detect health issues early and improve the overall well-being of their cattle.

## 1. Introduction

The health, welfare, and productivity of intensively housed cattle are profoundly influenced by their daily behavioral patterns. Time-budget allocations for fundamental states such as standing, lying, and feeding provide critical, non-invasive insights into an animal’s physiological and psychological status, serving as a vital biological barometer for assessing both individual well-being and the suitability of the group housing environment [1]. In modern dairy and beef production systems, where early intervention is paramount, behavioral changes often constitute the first, and sometimes only, sign of underlying health issues [2]. For instance, deviations in lying duration and frequency are strongly correlated with the onset of hoof pathologies and lameness, a major welfare and economic concern [3]. Similarly, a significant reduction in feeding time and altered feeding rhythms are recognized as early warnings for metabolic disorders like ketosis and subacute ruminal acidosis [4]. Consequently, the continuous and accurate monitoring of these core behaviors forms a cornerstone of preventive veterinary medicine and the successful implementation of precision livestock farming [2].

Despite its critical importance, traditional monitoring in many commercial operations still relies predominantly on manual observation and scheduled human patrols. While experienced caretakers can identify overt signs of distress, this approach is inherently limited by its episodic nature, high labor cost, and susceptibility to observer fatigue and inconsistency. It is fundamentally incapable of capturing the subtle, progressive shifts in daily time budgets that are crucial for the very early detection of health impairments [5]. The economic and welfare consequences of this limitation are well-documented [3]. For example, Ferronato et al. [6] demonstrated that a delay in diagnosing clinical mastitis by just one estrus cycle (approximately 21 days) led to an average milk production loss of 155–734 kg per lactation, with most of this reduction occurring before visible symptoms appeared. In the context of lameness, Roche et al. [3] observed that daily milk yield declined by an average of 0.7 kg as early as 4–6 weeks before a cow was visibly identified as lame (score ≥ 2), by which time many hoof lesions were already irreversible. Furthermore, the manual recording of behavioral data is intrinsically inefficient and prone to inaccuracy. As Grinter et al. [7] rigorously demonstrated, the test–retest reliability of manual logs for activities like feeding and rumination was significantly lower (correlation 0.62–0.68) than that achieved by automated sensor collars (correlation 0.92), underscoring the inadequacy of manual methods for meeting the demands of data-centric, precision livestock management.

In response to these shortcomings, the past decade has seen a surge in research into contact-based automated monitoring using wearable sensors. This line of inquiry has yielded various technological solutions, including systems that employ tri-axial accelerometers embedded in collars or ear tags to classify behavioral states [8,9,10,11,12], noseband pressure sensors to precisely detect jaw movements associated with feeding and rumination [13,14], and the integration of gyroscopes to enhance recognition accuracy [15]. While these sensor-based methods can be effective for individual animal monitoring, they present significant practical and economic barriers to sustainable, whole-herd adoption. The per-unit cost of procuring, maintaining, and periodically replacing sensors for every animal can be prohibitive, especially for large herds [2]. From an animal welfare perspective, the devices themselves may cause discomfort, pose a risk of injury, or alter the very behaviors they are intended to measure, while their physical attachment often necessitates frequent handling of the animals, inducing stress [1,16]. Moreover, from a data perspective, single-point sensing on an animal struggles to capture complex group interactions or spatial behaviors within the pen, limiting a holistic understanding of herd dynamics [17].

These inherent limitations of contact-based approaches have catalyzed a significant shift towards non-contact, vision-based analysis leveraging advanced deep learning techniques. This paradigm offers the promise of stress-free, scalable monitoring using standard farm cameras. Considerable progress has been made, with researchers developing sophisticated network architectures tailored to this task. For instance, Yang et al. [18] and Han et al. [19] proposed dual-stream and complex spatiotemporal models for recognizing cattle movement and aggressive behaviors, respectively, achieving high accuracy. Other studies have successfully adapted or enhanced established object detection architectures like YOLO and ResNet, or combined 3D Convolutional Neural Networks (C3D) with Long Short-Term Memory (LSTM) networks to model temporal information for classifying a broader range of behaviors [20,21]. A dominant and understandable trend across these state-of-the-art methods is the primary pursuit of maximizing recognition accuracy, which often leads to increased model complexity, parameter counts, and computational demands.

This prevailing focus, however, has created a critical and often overlooked implementation gap, particularly for the target application of affordable, continuous, and farm-wide monitoring. While these complex models may excel in controlled research settings or when deployed on powerful cloud servers, their high computational cost and large model sizes render them fundamentally unsuitable for deployment on the low-power, resource-constrained edge devices that represent the most economically viable and practical computing solution for the majority of farms, especially small and medium-sized enterprises [2]. The existing advanced solutions, therefore, risk being confined to proof-of-concept studies or relying on infrastructure that may not be available or reliable in typical agricultural environments, where real-time feedback is essential for timely intervention [22].

To bridge this gap and advance the sustainability of livestock production systems, there is a pressing need for monitoring solutions that are not only accurate and non-intrusive but also resource-efficient and economically accessible. Sustainable precision livestock farming hinges on technologies that minimize environmental impact, optimize resource use (including energy and computational resources), and remain viable for long-term adoption across diverse farm scales and economies [16]. Computer vision systems deployed on low-power edge devices align closely with these sustainability goals by reducing reliance on costly hardware, lowering energy consumption, and enabling proactive health management that can curtail production losses and unnecessary treatments.

To this end, this study proposes a computer vision-based system specifically engineered for end-to-end deployment in a pastoral setting [23]. We focus on the robust, automated recognition of three core, static behaviors—standing, lying, and eating—which constitute the foundational elements of a cattle’s daily time budget and serve as primary indicators for health and welfare assessment [4]. Our primary objective is not only to achieve high recognition accuracy but, more critically, to construct a model that is fundamentally deployable [3]. This involves a deliberate and integrated design philosophy that prioritizes a lightweight architecture, minimal computational overhead, and high inference speed from the outset, without compromising practical utility. The proposed model, based on an improved YOLO11n architecture, integrates several targeted enhancements, including advanced attention mechanisms and efficient feature fusion modules, to achieve this essential balance. By deploying such a pruned and optimized model directly on edge devices within the farm, this work aims to translate algorithmic advancements into a feasible, low-cost, and non-intrusive tool for veterinarians and farmers [1]. This technology empowers proactive herd health management by enabling the early detection of anomalies in behavioral time budgets, thereby contributing directly to enhanced animal welfare, optimized resource efficiency, and more sustainable farming practices [16].

## 2. Materials and Methods

### 2.1. Dataset Selection

The dataset utilized in this study was derived from a commercially sourced collection, the Korean Beef Cattle Farming Monitoring Dataset, hosted on the AiHub platform. Due to commercial confidentiality and licensing agreements, the raw dataset is not publicly distributable. However, for the purposes of scholarly review and to ensure the reproducibility of our experimental findings, a comprehensive description of the dataset’s composition and key characteristics is provided herein. This dataset contains annotations for three typical cattle behaviors: standing, lying down, and feeding. These behaviors represent the most common and fundamentally valuable actions for monitoring during the farming process. The dataset comprises 12,798 valid samples with original image resolution of 1920 × 1081. To enhance processing efficiency, all images were uniformly resized to 640 × 360 resolution for this study.

**Figure 1:**
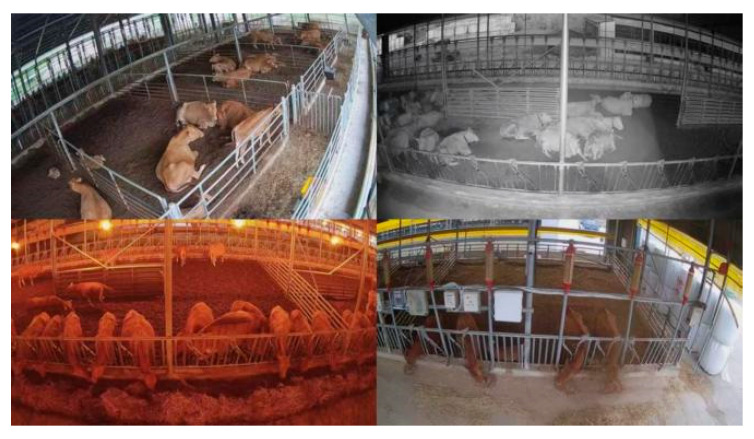
Example of a Farm Under Different Lighting Conditions.

To validate model performance, the dataset was split into training (10,698 images), validation (1100 images), and test (1000 images) sets at an 8:1:1 ratio. The sample label counts for each behavior category (standing, lying, feeding) are summarized in Table 1. This dataset exhibits the following key characteristics: First, it comprehensively covers common farm lighting scenarios, including daylight, artificial lighting, and infrared illumination. Second, it realistically incorporates complex interference scenarios such as mutual occlusion among cattle and barrier obstructions. Additionally, the dataset includes both sparse scenes with 4 to 8 cattle and dense group scenes with 10 to 20+ cattle, while also covering individuals at different growth stages, including adult cattle and calves, demonstrating broad scene coverage capability. A sample of the dataset is shown in Figure 1.

### 2.2. Experimental Environment and Parameter Settings

The experimental environment for this study utilizes the Ubuntu 22.04 operating system, based on the Python 3.12 programming language and PyTorch 2.51 deep learning framework, with GPU acceleration enabled via CUDA 12.4. Hardware configuration included: NVIDIA RTX 4090 GPU (24 GB VRAM), 16-core Intel Xeon Gold 6430 CPU, 120 GB RAM, along with a 30 GB system disk and 50 GB data disk.

After multiple rounds of experimental validation, the optimal parameter settings are as follows: training batch size (batchsize) 32, training iterations (epochs) 100, input image dimensions 640 × 640 pixels, weight decay coefficient 0.01, initial learning rate 0.01, momentum factor 0.937.

### 2.3. Cattle Behavior Recognition Methods

#### 2.3.1. Selection of Base Network Models

To construct an efficient and accurate cattle behavior recognition model, an appropriate base network architecture must first be selected. This study systematically compared multiple mainstream models within the YOLO series, including YOLOv3-tiny, YOLOv5, YOLOv8, YOLOv10, and YOLO11n. Their performance was comprehensively evaluated using the dataset developed in this research to determine the most suitable lightweight base network architecture for cattle behavior recognition tasks. The experimental comparison results are shown in Table 2. YOLO11n demonstrated the best overall performance, achieving a mAP@0.5 of 90.7%, significantly outperforming other models.

YOLO11n achieves an optimal balance between accuracy and efficiency. Its high recognition accuracy and low resource consumption characteristics lay a solid foundation for subsequent lightweight design and deployment. Therefore, this study selects YOLO11n as the base model for the cattle behavior recognition task.

#### 2.3.2. Incorporation of Multidimensional Collaborative Attention (MCA)

To address the challenges of inaccurate behavioral feature extraction and limited model discrimination in complex pasture environments, this study integrates a multidimensional collaborative attention mechanism into the deep architecture of an enhanced YOLOv11 backbone network. By synergistically optimizing the interaction logic between channel-wise and spatial-wise feature information, the model achieves adaptive focus on key behavioral regions—such as standing, lying, and feeding. This approach significantly enhances behavioral discrimination accuracy in dynamic pasture scenarios—such as varying light conditions, weed obstructions, and overlapping cattle—while preserving the model’s lightweight characteristics.

Let the input feature map be $F\in R^{C\times H\times W}$, where C represents the number of channels, carrying different types of cattle features (e.g., contour, texture); H is the spatial height of the feature map, and W is the spatial width; together they determine the spatial resolution of features, affecting the ability to capture local details. This mechanism processes dependency relationships across different feature dimensions through three parallel branches. In the feature aggregation stage, the features of each channel m∈{1,…,C} first undergo dual-path statistic extraction.

The first is the global average pooling operation, which calculates the average feature value of channel m across all spatial positions (i, j), where i and j are the height and width coordinates, respectively. This captures the global feature distribution of cattle behavior. The formula is:

(1) $$f_{m}^{avg}=\frac{1}{H\times W}\sum_{i=1}^{H}\sum_{j=1}^{W}f_{m}(i,j)$$

In Equation (1), $f_{m}(i,j)$ denotes the feature value of the m-th channel at spatial position (i,j), H×W is the total number of spatial pixels, and $f_{m}^{avg}$ represents the global average feature trend of channel m.

The second is the global standard deviation pooling operation, which captures the dispersion degree of the feature distribution, enhancing local features such as head movements or leg details when standing. The formula is:

(2) $$f_{m}^{std}=\sqrt{\frac{1}{H\times W}{\sum_{i=1}^{H}\sum_{j=1}^{W}\left(f_{m}(i,j)-f_{m}^{avg}\right)}^{2}}$$

In Equation (2), the symbols have the same meanings as above, and $f_{m}^{std}$ quantifies the feature dispersion, highlighting local details.

To fuse global and local features, learnable parameters are introduced for adaptive fusion. The formula is:

(3) $$\hat{F}=\frac{1}{2}\left(F^{avg}+F^{std}\right)+\alpha F^{avg}+\beta F^{std}$$

In Equation (2), α and β are learnable fusion coefficients (dynamically adjusted during training, e.g., increasing β when identifying feeding behavior to emphasize mouth details), $F^{avg}$ and $F^{std}\in R^{\left(C\times 1\times 1\right)}$ represent the mean statistic vector and standard deviation statistic vector for all channels, respectively, and $\hat{F}$ is the aggregated feature map of F. This design ensures the feature description retains both global contextual information and incorporates local detail features, providing effective input for cattle behavior discrimination.

#### 2.3.3. Convolution Layer Enhancements

In cattle behavior recognition scenarios, models must balance recognition accuracy with lightweight characteristics to suit edge computing devices (such as embedded terminals deployed in farms). While YOLO11n excels in feature extraction and detection efficiency as a mainstream object detection architecture, its standard convolutional operations suffer from high parameter counts and computational overhead, making it difficult to meet lightweight requirements. To address this, this study introduces the Deep Separable Convolution (DWConv) module at the critical downsampling stage to optimize YOLO11n’s feature extraction network.

DWConv decomposes standard convolutions into two steps, tailored to cattle behavior recognition requirements:

Depthwise Convolution: Differences in cattle behavior (such as body contours when standing or head posture when feeding) are manifested in spatial forms. This step applies C separate, $D_{K}\times D_{K}\times 1$ convolutional kernels to the C input channels, precisely extracting spatial features without redundant inter-channel computations, resulting in an output of C feature maps.

Pointwise Convolution: Distinguishing cattle behaviors requires the fusion of features from multiple channels (e.g., combining coat color features with posture features). This step uses M kernels of size 1×1×C to linearly combine the outputs from the depthwise convolution, adjusting the channel dimension to M, thereby providing support for behavior classification.

Considering the feature dimensions typical of cattle behavior recognition models(input feature map size $D_{F}\times D_{F}\times C$), the number of parameters required for a standard convolution is $D_{K}\times D_{K}\times C\times M$. In contrast, DWConv requires only $D_{K}\times D_{K}\times C+C\times M$ parameters. The parameter compression ratio is approximately $\frac{1}{N}+\frac{1}{D_{k}^{2}}$. This significantly reduces the storage and computational burden of YOLO11n on edge devices. Furthermore, the partitioned operation principle of DWConv aligns well with the feature extraction logic required for cattle behaviors. Even after the lightweight modification, the model maintains high recognition accuracy for behaviors such as standing, lying down, and feeding. This improvement provides crucial technical support for the practical deployment of the enhanced YOLO11n-based cattle behavior recognition model in farm settings.

#### 2.3.4. Improvement of Multi-Scale Feature Fusion

In the lightweight cattle behavior recognition task based on the improved YOLOv11n, significant variations in cattle scale (e.g., adult cattle vs. calves, distant vs. close-up views) and complex behavioral scenarios (e.g., variable postures like standing, lying down) necessitate an efficient multi-scale feature fusion module to enhance performance.

The neck network adopts BiFPN (Weighted Bi-directional Feature Pyramid Network) to replace the original PANet structure. By simplifying nodes and introducing learnable weights, BiFPN achieves efficient bidirectional fusion of cross-scale features, balancing expressive capability with lightweight requirements. Let the input feature set be ${\vec{P}}^{in}=\left(P_{l_{1}}^{in},P_{l_{2}}^{in},\ldots \right)$, and the output feature set be $P^{out}$, where $P^{in}$ is the set of multi-scale initial features of cattle output by the improved YOLOv11n backbone network. $P_{l_{i}}^{in}$ represents the feature map at level $l_{i}$ (a larger $l_{i}$ value corresponds to lower resolution but richer semantic information, such as the outline of distant cattle; a smaller $l_{i}$ value corresponds to higher resolution and more prominent details, such as leg movements of close-up cattle). The resolution of $P_{l_{i}}^{in}$ is $1/2^{l_{i}}$ of the input cattle image. During fusion, learnable non-negative weights activated by ReLU regulate the contribution of features. After unifying dimensions through Resize operations (bilinear upsampling or max pooling), normalized weighted calculation incorporating a small value ϵ (to prevent division by zero) and Depthwise Separable Convolution (Conv) are used to optimize the features. Taking the fusion at the 6th layer of BiFPN (corresponding to medium-scale cattle features) as an example, the intermediate feature $P_{6}^{td}$ in the top-down path (integrating high-level contour and mid-level body features) is generated by the formula:

(4) $$P_{6}^{td}=Conv(\frac{w_{1}\cdot P_{6}^{in}+w_{2}\cdot Resize(P_{7}^{in})}{w_{1}+w_{2}+\epsilon })$$

In Equation (4), $w_{1}$ and $w_{2}$ are the fusion weights for $P_{6}^{in}$ (the initial medium-scale feature at level 6) and $Resize(P_{7}^{in})$ (the upsampled high-level feature from level 7), respectively, and Conv performs feature extraction and dimensionality reduction. The output feature $P_{6}^{out}$ in the bottom-up path (further integrating low-level details) is generated by the formula:

(5) $$P_{6}^{out}=Conv(\frac{w_{1}^{\prime }\cdot P_{6}^{in}+w_{2}^{\prime }\cdot P_{6}^{td}+w_{3}^{\prime }\cdot Resize(P_{5}^{out})}{w_{1}^{\prime }+w_{2}^{\prime }+w_{3}^{\prime }+\epsilon })$$

In Equation (5), $w_{1}^{\prime }$, $w_{2}^{\prime }$ and $w_{3}^{\prime }$ are the weights for $P_{6}^{in}$, $P_{6}^{td}$ and $Resize(P_{5}^{out})$ (the upsampled low-level detail feature from level 5), respectively, and $P_{6}^{out}$ is the final output feature at level 6. Simultaneously, BiFPN uses fast normalized fusion instead of softmax to reduce computational complexity, adapting to lightweight requirements. After embedding BiFPN into the improved YOLO11n, its bidirectional information flow enhances the interaction between high and low-resolution features of cattle, and the dynamic weights reduce missed detections and false alarms. The use of depthwise separable convolution and fast normalized fusion decreases computational load, making it suitable for the computing power of edge devices deployed on farms. The iterative fusion enhances the feature robustness against interference like lighting variations and occlusions, ensuring high accuracy in cattle behavior recognition.

#### 2.3.5. Improvements to Static Cattle Behavior Recognition Methods

Given that the dataset primarily consists of images capturing static behaviors such as standing, lying down, and feeding, this study balances lightweight model deployment with recognition accuracy by this study adopts GELAN as the core feature fusion architecture for enhancing YOLO11n. Feature transformation employs GELAN-based RepNCSPELAN4 and C3k2 modules, utilizing cross-stage partial network structures and reparameterization techniques to improve multi-scale feature integration efficiency. This architecture combines CSPNet’s gradient diversion advantages with ELAN’s feature aggregation capabilities. Through refined gradient path planning, it preserves critical static behavior features (e.g., recumbent body posture, foraging head angle) while reducing computational overhead for embedded farm devices, enabling precise behavior extraction in static scenarios.

The RepNCSPELAN4 module employs cross-cascade splitting to focus on details like standing torso contours and relative positions between feeding heads and feed, reducing redundant background interference. RepConvN parameter transformation lowers inference latency to support 24 h real-time monitoring. SPPELAN multi-scale pooling captures static behavior differences across cattle of varying body types, addressing low identification accuracy in feeding zones.

At the feature fusion level, GELAN’s multi-branch topology prevents loss of subtle static behavioral features (e.g., standing leg joint angles, recumbency body extension) in deep layers. Its learnable gating mechanism adaptively adjusts branch weights based on behavioral complexity—reducing computational load for simple actions like standing/lying while enhancing fusion accuracy for complex behaviors like feeding—to accommodate diverse static behaviors across datasets. This dynamic weighting strategy constitutes GELAN’s core innovation, enhancing the model’s generalization capability for static behaviors while balancing lightweight architecture with recognition accuracy.

Additionally, grouped convolutions reduce parameters to minimize device memory usage. Channel reordering ensures feature correlation between head, torso, and limbs in static behaviors (e.g., coordinated head-torso postures during feeding). Residual connections mitigate gradient vanishing, guaranteeing stable feature extraction in low-light and occlusion scenarios. These designs enable GELAN to maintain efficient gradient propagation while reducing parameters and power consumption, paving the way for edge deployment.

#### 2.3.6. Improved Daily Behavior Recognition Model for Cattle

**Figure 2:**
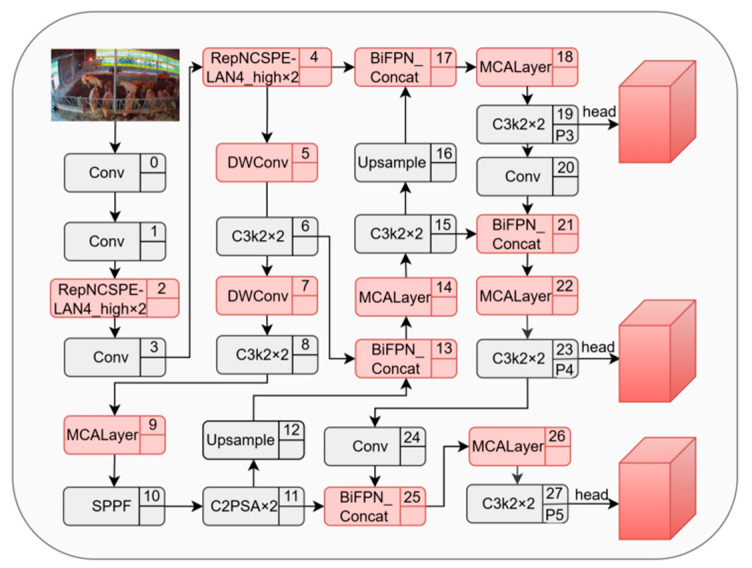
Network Architecture Diagram of the Improved YOLO11n Model.

Based on the preceding analysis, this study systematically enhanced the YOLO11n model to construct a lightweight detection model integrating multidimensional collaborative attention mechanisms, separable convolutions, bidirectional feature pyramid networks, and a universal efficient layer aggregation network. The overall architecture of the improved YOLO11n model is shown in Figure 2. Gray-background rectangles denote unchanged original YOLO11n modules, while red-background rectangles represent introduced improvement modules. Together, they constitute the complete improved YOLO11n model. The improved model incorporates multiple optimizations in its network architecture:

This enhanced model achieves lightweight processing through DWConv, strengthens feature selectivity with MCA, and optimizes feature fusion and extraction pathways using BiFPN and GELAN, thereby constructing an efficient and precise cattle behavior recognition model.

### 2.4. Model Pruning

In the context of cattle behavior recognition for precision farming, while YOLO11n is already a lightweight model capable of adapting to the basic computing power of edge devices and identifying cattle behaviors such as standing, lying down, and feeding, there remains room to reduce its parameters and computational costs. Further lightweighting is needed to enhance deployment flexibility. Compared to mainstream lightweight methods:—Model quantization risks losing behavioral feature details, reducing accuracy for similar actions;—Knowledge distillation relies on teacher models and suffers from overfitting with limited farming data, while structural redesign requires lengthy development cycles;—Transfer learning faces feature adaptation bias due to data distribution differences between source and target domains, necessitating additional domain adaptation tuning that increases deployment complexity. Pruning algorithms directly eliminate redundant parameters, preserving superior behavioral feature extraction capabilities at equivalent compression rates. Requiring no additional dependencies or complex designs, they better align with livestock farming needs, making them the preferred lightweight approach in this study.

While traditional weight-amplitude-based pruning is simple and efficient, it suffers from unreasonable inter-layer pruning ratio allocation, potentially impairing YOLO11n’s ability to extract cattle behavioral features. To address this, this study introduces the LAMP (Layer-Adaptive Magnitude-based Pruning) pruning algorithm for secondary lightweight optimization of YOLO11n. This algorithm integrates hierarchical pruning with an adaptive mechanism, focusing on minimizing pruning distortion. It treats pruning at each layer of YOLO11n as a perturbation to the operator output, optimizing the inter-layer pruning ratio through a unified weight importance scoring system. The scoring formula is:

(6) $$score(u;W):=\frac{(W[u]{)}^{2}}{\sum_{v\geq u}(W[v]{)}^{2}}$$

In Equation (6), W represents the weight tensor, u and v are weight indices, and the weights are sorted in ascending order of magnitude, meaning that if u<v, then |W[u]|≤|W[v]|. The numerator $(|W[u]|{)}^{2}$ represents the squared magnitude of the current weight, and the denominator $\sum_{v\geq u}(W[v]{)}^{2}$ is the sum of squared magnitudes of all weights in the current layer that are not less than the current weight. This score dynamically reflects weight importance without requiring hyperparameter tuning.

The process of applying LAMP to YOLO11n can be illustrated with a matrix representation:

(7) $$\left[\begin{array}{c} u_{1}\\ u_{2}\\ u_{3}\\ \ldots \end{array}\right]\left[\begin{array}{c} v_{1}\\ v_{2}\\ v_{3}\\ \ldots \end{array}\right]⟶\left[\begin{array}{c} \frac{u_{1}^{2}}{u_{1}^{2}}\\ \frac{u_{2}^{2}}{u_{1}^{2}+u_{2}^{2}}\\ \frac{u_{3}^{2}}{u_{1}^{2}+u_{2}^{2}+u_{3}^{2}}\\ \ldots \end{array}\right]\left[\begin{array}{c} \frac{v_{1}^{2}}{v_{1}^{2}}\\ \frac{v_{2}^{2}}{v_{1}^{2}+v_{2}^{2}}\\ \frac{v_{3}^{2}}{v_{1}^{2}+v_{2}^{2}+v_{3}^{2}}\\ \ldots \end{array}\right]⟶\left[\begin{array}{cc} \frac{u_{1}^{2}}{u_{1}^{2}} & \frac{v_{1}^{2}}{v_{1}^{2}}\\ \frac{u_{2}^{2}}{u_{1}^{2}+u_{2}^{2}} & \emptyset \\ \emptyset & \emptyset \\ \ldots & \ldots \end{array}\right]$$

In Equation (7), the left part represents the sorted weight magnitudes for each layer of YOLO11n, the middle part represents the LAMP scores, and the right part represents the global pruning result. This algorithm automatically assigns appropriate sparsity levels to each layer of YOLO11n. While further compressing model parameters and computational load, it avoids the decline in cattle behavior recognition accuracy caused by unbalanced pruning, facilitating the stable deployment of the model on edge devices with even lower computational capabilities.

## 3. Results

### 3.1. YOLO11n Model Improvement Ablation Study

**Figure 3:**
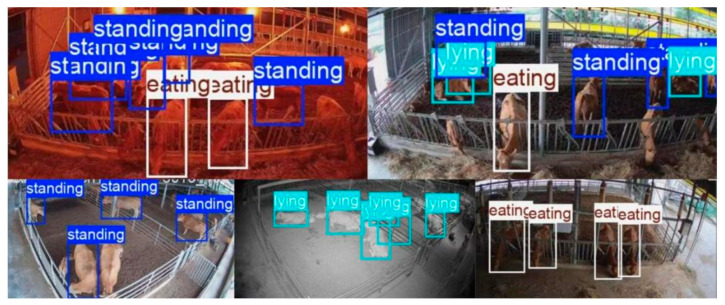
Cattle Behavior Recognition Effect.

To validate the effectiveness of each enhancement module, this study conducted ablation experiments to determine their contribution rates to the recognition of cattle daily behaviors. The experimental results are shown in Table 3, and the recognition performance is illustrated in Figure 3.

### 3.2. Model Robustness Testing Experiments

Lighting conditions in actual aquaculture environments are highly variable. To validate the robustness of the improved YOLOv11n model under different lighting conditions, this study conducted tests in three typical scenarios: daylight, artificial lighting, and infrared light. As shown in Table 4.

### 3.3. Pruning Experiment Results for Different Acceleration Ratios

To balance model efficiency and accuracy, pruning experiments were conducted on the improved YOLO11n model using different acceleration ratios. The experiments demonstrated that at a 1.50x acceleration ratio, the model significantly improved computational efficiency while maintaining high accuracy, representing the optimal pruning scheme.

As shown in Table 5, the original model achieved a mAP@0.5 of 92.0% at an acceleration ratio of 1.00. When the acceleration ratio was increased to 1.50, the number of parameters decreased from 2.38 × 10^6^ to 1.06 × 10^6^, a reduction of 55.5%; computational load decreased from 9.7 GFLOPS to 6.3 GFLOPS, a reduction of 35.1%; and the weight file was compressed to 2.4 MB. At this point, the model maintained 90.5% mAP@0.5 and 68.8% mAP@0.5–0.95, with accuracy loss controlled within a reasonable range. Simultaneously, the frame rate increased by 31.2% to 153.9 FPS. When the acceleration ratio was further increased to 1.75x, the mAP@0.5 dropped to 88.3%. Beyond 2.00x, model performance deteriorated sharply, with the mAP@0.5 plummeting to just 29.8%, indicating that excessive pruning leads to model failure.

Comprehensive experimental results show that a 1.50x acceleration ratio achieves the optimal balance between accuracy retention and efficiency improvement. It not only meets real-time requirements but also maintains a detection accuracy rate above 90%, making it suitable for real-time monitoring needs in practical aquaculture scenarios.

### 3.4. Pruning Results Analysis

**Figure 4:**
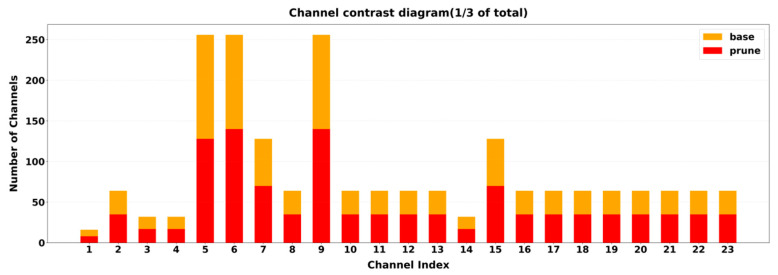
Channel Comparison Chart.

Based on the aforementioned experiments, the optimal solution was determined to be a 1.50x acceleration ratio combined with the LAMP strategy. In terms of pruning effectiveness, the base model had 5728 total channels. After LAMP algorithm pruning, the total channels were reduced to 3113, achieving an overall compression ratio of 45.65%. The layer with the highest pruning intensity reduced 128 channels. Channel pruning comparisons are shown in Figure 4 (to clearly illustrate major changes and effects, channels with low pruning rates and those retaining only one-third of their original channels were excluded). The orange bars represent the original channel count, while the red bars indicate the post-pruning channel count. It is evident that the LAMP algorithm exhibits differentiated pruning rates across different network layers, demonstrating its ability to adaptively identify and retain important feature channels. This effectively reduces redundancy, achieving lightweight optimization while preserving the model’s expressive capability.

The above results validate the effectiveness of the LAMP strategy in the YOLOv11n cattle behavior recognition model. As shown in Table 6, this approach not only significantly reduces model parameters and computational overhead but also maintains good detection accuracy and real-time performance.

## 4. Discussion

This study developed a lightweight recognition model based on an improved YOLOv11n architecture for monitoring key cattle behaviors. The experimental results demonstrate that the proposed model achieves an optimal balance between high recognition accuracy and minimal computational resource consumption, rendering it highly suitable for deployment on edge devices within farm environments.

### 4.1. Interpretation of Findings and Practical Benefits

The systematic integration of enhancement modules—MCA, DWConv, BiFPN, and GELAN—collectively contributed to a progressive performance improvement, as evidenced by the ablation study presented in Table 3. The incorporation of the Multidimensional Collaborative Attention mechanism enhanced the model’s capability to focus on discriminative regions associated with specific behaviors, such as head posture during feeding or body contours during lying. This attribute is particularly crucial in complex scenes involving occlusions or varying illumination, a finding consistent with prior research underscoring the value of multidimensional attention mechanisms in agricultural vision tasks [24]. The subsequent integration of the Bidirectional Feature Pyramid Network and the Generalized Efficient Layer Aggregation Network further strengthened the model’s multi-scale feature fusion and gradient flow efficiency [25]. This synergistic enhancement culminated in a significant improvement of the overall mAP@0.5 from a baseline of 90.7% to 92.0% for the final improved model, which achieved high recognition rates of 91.2% for standing, 91.0% for lying, and 93.9% for eating.

A central design objective was to maintain high accuracy while achieving model lightweightness. The strategic replacement of standard convolutions with Depthwise Separable Convolutions in the backbone network was instrumental in this regard. This modification reduced the model parameters from 2.81 × 10^6^ to 2.38 × 10^6^ and computational load from 10.4 GFLOPS to 9.7 GFLOPS, while the mAP@0.5 was further elevated to 92.0%. This outcome confirms the efficacy of depthwise separable convolutions for constructing efficient models compatible with edge computing paradigms [22].

### 4.2. Model Lightweighting and Practical Implications

The application of the Layer-Adaptive Magnitude-Based Pruning algorithm proved highly effective for structural model compression, representing a critical step towards practical deployment [26]. The pruned model was compressed to 1.06 × 10^6^ parameters and 2.4 MB in size, with a computational cost of only 6.3 GFLOPS. This substantial reduction—representing a 55.5% decrease in parameters and a 35.1% reduction in GFLOPS compared to the unpruned improved model—was achieved while retaining a mAP@0.5 of 90.7%, corresponding to a minimal accuracy loss of only 1.3%. This degree of lightweighting facilitates real-time, multi-point monitoring on low-cost hardware, a decisive advantage for small and medium-sized farms operating with limited budgets.

The model’s robustness was further validated under different lighting conditions, as detailed in Table 4. It maintained strong performance under daylight with a mAP@0.5 of 91.7% and artificial light with 91.1%, demonstrating its potential for continuous, round-the-clock operation in practical settings. A performance decline was observed for the eating behavior under infrared light, where mAP@0.5 dropped to 81.0%, likely due to the lack of color and reduced texture details impairing the discrimination of subtle head and mouth postures [17]. Nonetheless, the system’s overall balance of deployability and maintained accuracy represents a significant advancement over many existing models that prioritize accuracy at the expense of practicality.

### 4.3. Limitations and Future Research

Despite the promising results, certain limitations should be acknowledged. First, the model was trained and validated on a dataset containing three primary static behaviors. While these behaviors serve as fundamental indicators of cattle health and welfare, future work should expand the behavioral repertoire to include dynamic actions such as walking, drinking, and ruminating, as well as critical abnormal behaviors like lameness or aggression. Second, the current model operates on single images. Incorporating temporal information by extending the framework to video sequence analysis could further improve recognition robustness and enable the prediction of behavior patterns. Finally, while the model shows robustness to various lighting, its performance could be enhanced by training on more extensive datasets encompassing a wider variety of environmental challenges, such as heavy occlusion, diverse weather conditions, and different cattle breeds.

In conclusion, this research provides a viable and efficient solution for real-time cattle behavior monitoring. The proposed model effectively balances accuracy and efficiency, offering a practical tool to support precision livestock farming. Future efforts will focus on expanding behavioral classes and integrating temporal modeling to develop a more comprehensive monitoring system.

### 4.4. Veterinary and Animal Welfare Implications

The capacity for real-time, automated monitoring of core behaviors like standing, lying, and feeding holds profound implications for advancing veterinary care and animal welfare standards in cattle production [2]. This technology directly translates behavioral ecology into actionable health insights. Changes in the duration, frequency, or timing of these behaviors are among the most sensitive and early indicators of health disorders [1]. For instance, a sustained increase in lying time often signals the pain and discomfort associated with lameness or hoof lesions, while a decline in feeding activity is a recognized precursor to metabolic issues such as ketosis or systemic illness [3,4].

The proposed system enables a paradigm shift from reactive treatment to proactive, preventive husbandry. By providing continuous, non-intrusive surveillance, it facilitates early intervention at the most tractable stages of a disease, which is critical for mitigating animal suffering, preventing the progression of conditions like mastitis, and reducing the need for antimicrobial treatments [3]. Moreover, the non-contact nature of the system aligns perfectly with the core principles of low-stress animal handling and precision livestock farming [16]. It eliminates the potential discomfort, stress, and injury risks associated with wearable sensors, thereby safeguarding animal welfare throughout the monitoring process itself.

Ultimately, this technology serves as a force multiplier for veterinary and farm management. It empowers stakeholders to make data-driven decisions based on objective, continuous behavioral data rather than intermittent observations. This enhances the efficiency and effectiveness of herd health management, allowing for the early identification of at-risk individuals and the optimization of resource allocation for care. By making sophisticated monitoring scalable and affordable, the system offers a practical pathway to elevate welfare benchmarks and promote more sustainable and ethical cattle production practices [17].

## 5. Conclusions

This study proposes a lightweight cattle behavior recognition method based on an improved YOLO11n architecture. By strategically incorporating innovative modules such as MCA, DWConv, BiFPN, and GELAN, it achieves an optimal balance between model compactness and recognition accuracy. Experimental results demonstrate that the improved model achieves high recognition accuracies for three typical cattle behaviors, with performance improvements over the baseline model. After pruning with the LAMP algorithm, the model was significantly compressed to 1.06 × 10^6^ parameters and 2.4 MB in size, while maintaining a mAP@0.5 of 90.7%.

The primary technical contribution of this work lies in delivering a high-accuracy model that is amenable to deployment on low-cost, resource-constrained edge devices. This attribute of low-demand computational infrastructure makes it a practical tool for veterinary and farm management, enabling early detection of health anomalies and supporting animal welfare through continuous, non-invasive monitoring. By lowering the barrier to adopting automated monitoring technology, the proposed system holds particular promise for farming operations with limited capital for advanced computing hardware. Furthermore, by facilitating early disease intervention and improving management efficiency, this approach can contribute to more sustainable operational practices by helping to optimize resource use and reduce losses.

## Figures and Tables

**Table 1:** Number of Tags for Each Category of Cattle Behavior.

Behavior Category	Number of Tags		
	Training Set	Validation Set	Test Set
standing	37,232	3709	3966
lying	22,604	2719	2040
eating	15,921	1491	1128

**Table 2:** Comparative Experiments of Basic Network Models (mAP@0.5: mean Average Precision at IoU threshold 0.5; params: number of parameters; GFLOPS: Giga Floating-point Operations Per Second; weights: model file size in Megabytes).

Model	Precision	Recall	mAP@0.5/%	mAP@0.5–0.95/%	Params	GFLOPS	Weights	mAP@0.5/%		
								Standing	Lying	Eating
YOLO11n	85.7	83.3	90.7	68.7	2.59 × 10^6^	6.4	5.2	89.9	88.9	93.3
Yolov10	83.3	85.2	90.6	69.4	2.70 × 10^6^	8.4	5.3	90.1	88.7	93.2
Yolov8	82.2	85.5	90	68.4	3.01 × 10^6^	8.2	5.3	89.8	87.6	92.7
Yolov5	83.9	83.3	89.5	66.8	2.50 × 10^6^	7.2	10.7	89.6	85.9	93
YOLOv3-tiny	79.6	83.3	89.6	66.7	1.212 × 10^7^	18.9	24.4	85.6	87.7	95.6

**Table 3:** Ablation Experiments for the Improved YOLO11n Model (mAP@0.5: mean Average Precision at IoU threshold 0.5; params: number of parameters; GFLOPS: Giga Floating-point Operations Per Second; weights: model file size in Megabytes).

Model	mAP@0.5/%	Param	GFLOPS	Weights	mAP@0.5/%		
					Standing	Lying	Eating
YOLO11n	90.7	2.59 × 10^6^	6.4	5.2	89.9	88.9	93.3
YOLO11n+MCA	90.9	2.62 × 10^6^	6.6	5.3	89.9	89.7	93.1
YOLO11n+MCA+BiFPN	91.2	2.28 × 10^6^	6.3	5.3	90.4	89.4	93.6
YOLO11n+MCA+BiFPN+GELAN	91.9	2.81 × 10^6^	10.4	10.7	91.2	90.6	93.8
YOLO11n+MCA+BiFPN+GELAN+DWConv	92.0	2.38 × 10^6^	9.7	4.9	91.2	91.0	93.9

**Table 4:** Experiments under Different Light Conditions (mAP@0.5: mean Average Precision at IoU threshold 0.5; params: number of parameters; GFLOPS: Giga Floating-point Operations Per Second; weights: model file size in Megabytes).

Light	Images	mAP@0.5/%	Param	GFLOPS	Weights	mAP@0.5/%		
						Standing	Lying	Eating
Day_light	631	91.7	2.37 × 10^6^	9.6	4.9	90.8	90.0	94.3
Lamp_light	41	91.1	2.37 × 10^6^	9.6	4.9	88.5	90.0	94.8
Infrared_light	268	88.5	2.37 × 10^6^	9.6	4.9	92.6	92.0	81.0

**Table 5:** Pruning Experiments at Different Acceleration Ratios (mAP@0.5 and mAP@0.5:0.95: mean Average Precision at single and multiple IoU thresholds, respectively; params: number of parameters; GFLOPS: Giga Floating-point Operations Per Second; weights: model file size in Megabytes; FPS: Frames Per Second).

Speed-Up	mAP@0.5/%	mAP@0.5–0.95/%	Param	GFLOPS	Weights	FPS
1.00	92.0	72.2	2.38 × 10^6^	9.7	4.9	117.3
1.25	90.7	69.7	1.55 × 10^6^	7.6	3.5	145.0
1.50	90.5	68.8	1.06 × 10^6^	6.3	2.4	153.9
1.75	88.3	64.5	0.76 × 10^6^	5.4	1.8	152.8
2.00	86.4	61.1	0.45 × 10^6^	4.8	1.3	158.0
2.25	29.8	12.9	0.29 × 10^6^	4.4	1.0	169.3
2.50	29	12.2	0.29 × 10^6^	4.4	1.0	167.9

**Table 6:** Pruning Results (mAP@0.5: mean Average Precision at IoU threshold 0.5; params: number of parameters; GFLOPS: Giga Floating-point Operations Per Second; weights: model file size in Megabytes; FPS: Frames Per Second).

Algorithm	mAP@0.5/%	mAP@0.5/%			Param	GFLOPS	Weights	FPS
		Standing	Lying	Eating				
Lamp	90.7	89.9	88.8	92.9	1.06 × 10^6^	6.3	2.4	153.9

## Data Availability

The data presented in this study are available on request from the corresponding author. Due to commercial confidentiality and licensing agreements, the raw dataset is not publicly distributable.
